# Clinical Features and Severity of Leptospirosis Cases Reported in the Hawke's Bay Region of New Zealand

**DOI:** 10.1155/2021/5567081

**Published:** 2021-07-06

**Authors:** Paul Sellors, Rebecca F. Watson, Rachel Bate, Gemma L. Bentham, Kathryn Haigh

**Affiliations:** ^1^Gerontology and Stroke Medicine, Southmead Hospital, Bristol, UK; ^2^Tropical and Infectious Diseases Unit, Royal Liverpool and Broadgreen University Hospitals Trust, Liverpool, UK; ^3^Nightingale Valley Practice, Bristol, UK; ^4^Obstetrics and Gynaecology, Royal United Hospitals Bath NHS Foundation Trust, Bath, UK; ^5^Institute of Infection, Veterinary and Ecological Sciences, University of Liverpool, Liverpool, UK

## Abstract

**Aims:**

To record demographics, symptoms, signs, and laboratory features of confirmed leptospirosis cases in the Hawke's Bay area of New Zealand to aid clinicians in diagnosis and recognition of severity.

**Methods:**

Review of suspected leptospirosis cases referred to the reference laboratory from hospitals in the Hawke's Bay region between March 2003 and March 2012. Inclusion criteria were IgM positivity and diagnosis confirmed with either polymerase chain reaction (PCR) or microscopic agglutination test (MAT). A retrospective systematic review of case notes was completed for demographic and laboratory data.

**Results:**

Forty-three cases were included. Most common presenting symptoms were pyrexia (93%), myalgia, and headache (both 86%). 93% of patients worked in the farming or meat industries. The most common biochemical abnormalities were elevated CRP (100%) and abnormal urinalysis (93%). There was no difference in disease severity between icteric and anicteric patients. Compared to other studies, patients in New Zealand have less severe disease.

**Conclusion:**

Contrary to popular understanding, this study has not found icteric leptospirosis to be related to more severe disease. Anicteric leptospirosis should be a differential diagnosis in patients presenting with pyrexia, myalgia, and headache who have elevated CRP and abnormal urinalysis.

## 1. Introduction

Worldwide, leptospirosis is the most common zoonotic disease [1]. It has been identified by the World Health Organization as a neglected disease of increasing importance [2]. Global incidence rates range dramatically from 0.1-1 per 100,000 per year in temperate climates to 10–100 per 100,000 per year in tropical areas [3]. This may grossly underestimate incidence in temperate areas, with studies citing leptospirosis as a cause in 20–40% of undifferentiated pyrexia [2, 4]. This was reiterated in a New Zealand study that diagnosed leptospirosis in 15% of patients with undifferentiated fever [5]. In New Zealand, the incidence is 2.5–8.0 per 100,000 per year, one of the highest rates amongst higher income countries [6–9].

Leptospirosis is caused by *Leptospira* spp.—bacteria of the phylum spirochetes. Infection is caused by contact with urine of carrier animals via abrasions, mucosal, or close animal contact. The main animal reservoirs are rats and livestock, although domestic animals are also recognised as carriers. *Leptospira* spp. survive for 3–7 weeks in contaminated environmental fluid reservoirs. Leptospirosis infection shows seasonal variation with increased transmission during warmer and more humid conditions [1, 10]. Infection risk is associated with occupation through exposure to animal urine and with recreational activities such as hiking and watersports [1].

Clinical manifestations range dramatically from a mild, flu-like illness through to fulminant hepatorenal failure. A wide spectrum of haemorrhagic complications are reported, from mild conjunctival suffusion to pulmonary haemorrhage [10].

Infection is identified serologically. Diagnosis in New Zealand is often through an initial immunological screening test using enzyme-linked immunosorbent assay (ELISA) for IgM antibodies. IgM positivity is a useful screening test but cannot be used to confirm the diagnosis due to crossreactivity with other conditions [11]. Confirmatory testing is undertaken with MAT or blood, urine, or CSF PCR [3]. The New Zealand Ministry of Health defines a confirmed case as a four-fold or greater rise between initial and convalescent samples or a single high antibody titre of >400 in the MAT [11]. Probable causative serovar is identified by MAT.

Leptospirosis cases may be underreported due to the nonspecific presentation and often slow diagnostic confirmation. However, with worldwide mortality from leptospirosis quoted between 5 and 30%, [3, 10] a high index of suspicion is required to prevent serious harm. Treatment with antibiotics remains controversial [12]. It has been suggested that earlier intervention, often supportive, may prevent serious sequelae [13, 14].

## 2. Methods

Ethical approval was granted from New Zealand Health Research Council (reference 12/02/102).

Data were collected on tests referred to the leptospirosis reference laboratory from all hospitals in the Hawke's Bay region between March 2003 and March 2012 (*n* = 702). Only cases with IgM antibody or PCR positivity were included (*n* = 179). These results were filtered for all confirmed cases, defined for the purpose of this study as initial MAT >800 (understood to be the national reference level for the seroprevalence of the area at time of data collection) or four-fold increase between initial and convalescent samples, or PCR positivity (*n* = 43).

Case notes were retrospectively systematically reviewed for demographic, clinicopathological, and outcome data (Tables 1–4). Results were compared with previous evidence from the literature, and icteric and anicteric patients were compared for features of severity including ICU admission and need for haemofiltration or vasopressor support. Icterus was defined as a serum bilirubin of >20 *μ*mol/L.

Formal statistical analysis was not carried out due to the small sample size.

## 3. Results

Baseline demographics for the 43 cases included in the study are shown in Table 1. The most common occupation was agriculture, 93% of patients presenting with leptospirosis worked in farming and meat industries. Patients were predominantly male (81.4%), of middle age (55.8%), and had no past medical history.

Most patients (73%) presented first to their GP and 21% of these had already been initiated on antimicrobial therapy. On average patients were unwell for six days prior to presentation (range 2–14 days).

Patients presented with a range of symptoms (Table 2), the most frequent being fever (93%) followed by headache and myalgia (both 86%).

There were a variety of laboratory sample abnormalities noted (Table 3). Most frequent was a raised CRP, a nonspecific marker for inflammation. Renal injury was commonly reported; 63% of patients had elevated creatinine values with 30% of patients meeting the definition for acute kidney injury (AKI, elevation in serum creatinine >1.5 times baseline). The majority of patients had abnormal urinalysis (88% proteinuria and 74% haematuria).

Icterus (serum bilirubin >20 *µ*mol/L) was identified in 40% patients with 60% defined as anicteric. Important markers of severity have been compared for the two groups (Table 4). There were no cases of fulminant hepatic failure, death, or requirement for intubation and ventilation. The length of hospital stay was similar between the two groups, but a higher percentage of patients in the anicteric group required admission to ICU, haemofiltration, and vasopressor support.

At presentation, 35% patients had normal observations, classified as systolic blood pressure >100 mmHg, heart rate <100 beats per minute, and respiratory rate <20 breaths per minute. Of the 28% of patients admitted to ICU, only one had normal observations on admission.

Admission chest radiographs were completed for eleven (26%) cases. Five were normal. The remaining films showed a range of abnormalities with no clear unifying features: cardiomegaly (*n* = 1), small areas of minimal atelectasis (*n* = 2), increased vascular markings (*n* = 1), and peribronchial thickening (*n* = 2). An electrocardiogram was carried out in ten (23%) cases, reported as normal (*n* = 3), sinus tachycardia (*n* = 5), and atrial fibrillation with fast ventricular response (*n* = 2).

All 43 cases were IgM ELISA positive and had their diagnoses confirmed with either MAT or PCR. Initial MAT was diagnostic in 4 (9%) of samples; all other sample sets demonstrated a four-fold increased between initial and convalescent samples. Of the 26 cases where at least one sample was sent for PCR testing, 20 (77%) were positive, 12 (60%), 6 (30%), and 2 (10%) on plasma, urine, or both PCRs, respectively.

**Figure 1:**
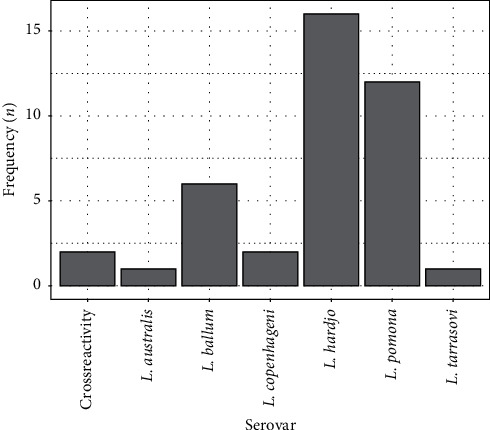
Serovars of leptospira found in the study population.

Samples were run for serovar (sv) identification in 95% cases at the reference laboratory, and six different sv were reported (Figure 1). One sample was negative and two cases were unable to be determined owing to crossreactivity. Three species of *Leptospira* were reported: *Leptospira interrogans* (sv *L. australis* and *L. copenhageni*), *L. borgpetersenii* (sv *L. ballum, L. hardjo,* and *L. tarrasovi*), and *L. interrogans* (sv *L. australis*). The most common sv seen in this dataset were *L. hardjo* and *L. pomona*.

Most patients received more than one antimicrobial. The most common prescriptions were of penicillins (62%) and doxycycline (57%). When stepping down or changing antimicrobial therapy, practitioners most commonly changed to doxycycline.

## 4. Discussion

### 4.1. Demographics

Occupation was the main risk factor for leptospirosis identified, with 93% of patients working in farming or meat processing industries. One case was linked to recreational activities. The link with the cattle industry is a consistent finding within studies in New Zealand [9]. The national reporting office listed meat processing and agricultural work as responsible for 76.9% of cases [8]. These findings contrast with other high income countries. Historically, occupation was the predominant risk factor in the majority of European cases, but more recent studies suggest that recreation—most notably watersports—and impoverished housing now play equally important roles [18]. In Germany, 30% of cases were linked to occupational exposure, 30% recreational and 37% residential [18]. In a Hawaiian study 41% of cases were occupational and 43% recreational [16].

Age, gender, and ethnicity in these data mirror the demographics of the workforce in the agricultural industries [8]. The most frequent serovars found in this study are in keeping with those most frequently isolated from cattle reservoirs and are consistent with previous epidemiological studies in New Zealand [6, 8]. *L. pomona* and *L. hardjo* are strongly associated with pigs and cattle, respectively [6, 8, 10]. The next most common, *L. ballum*, is predominantly associated with rodents, although cattle can act as a secondary reservoir. The prevalence of this serovar has been increasing and may represent contamination of livestock feed by foraging rodents [6, 19].

### 4.2. Presentation

Leptospirosis is typically thought to consist of two phases spanning 7–14 days: an initial febrile illness followed by an immune phase [1]. In this cohort no clear biphasic illness was described. However, most patients had presented to their GP prior to hospital presentation; thus, the biphasic element may have been missed. The average number of days of illness prior to presentation was six days, fewer than previously observed in other studies.

Patients in this cohort reported a wide range of nonspecific symptoms, in keeping with previous studies [5, 16, 17]. Compared with data from other countries [15–17], the most marked difference is that jaundice is much less frequently observed in New Zealand, 9% compared to 95% and 72% in patients in Barbados [17] and China [15], respectively. The unifying features in this cohort were the presence of fever, high CRP, and abnormal urinalysis. Headache, myalgia, and nausea were also common. The three patients who were apyrexial on admission all developed pyrexia later in the disease course.

### 4.3. Myalgia

Myalgia is known as a hallmark of leptospirosis and is typically described as localising to the back and legs [20, 21]. Histologically, there is focal necrosis of muscle fibres with a corresponding mild increase in creatinine kinase (CK); rhabdomyolysis is rare [1, 10]. Consistent with worldwide trends, most patients in this series reported myalgia that, when specified, commonly affected the back and legs. Only 36% patients had an elevated CK.

### 4.4. Headache

Headache is commonly reported in leptospirosis, often described as severe and associated with vomiting. Patients may present with impaired consciousness in the early phase followed by meningitic features in the quarter of cases during the immune phase [1]. Lumbar puncture may reveal elevated opening pressures and pleocytosis. In this series, headache was a common presentation. Meningitic features were described in 25% of those reporting headache, all at presentation and typically nonsevere. The mean age of patients reporting headache was 36 years, lower than the previously described 43 years. [1]. Impairment of consciousness, a late-stage feature of the disease, was absent in our dataset. This may be due to earlier presentation to hospital in this cohort with appropriate supportive treatment reducing the risk of deterioration.

### 4.5. Ocular

Conjunctival suffusion, particularly when paired with icteric sclera, has been described as pathognomonic for severe leptospirosis or “Weil's disease” [1]. The aetiology of ocular signs is unclear. Although small numbers of spirochetes are found within the eye during infection, ocular involvement and uveitis can present after acute infection, suggesting a potential autoimmune cause [1, 10]. In our sample, there were few patients with ocular symptoms or signs.

Prospective studies tend to report ocular signs more frequently than retrospective reviews; there could have been failure to record the sign rather than lower incidence. However, a prospective New Zealand study reported low incidence of ocular signs, [5] suggesting that it may be less common than in other countries. Given the postulated autoimmune aetiology, this could be due to host factors rather than infecting organism.

### 4.6. Renal and Hepatic

Renal dysfunction is the main organ failure associated with leptospirosis. Histologically, there are spirochetes in the renal tubules, interstitial nephritis, and glomerular damage with tubular necrosis [1, 22]. The aetiology is not fully known, but is likely to be a combination of direct toxic injury, immune-mediated responses, and circulatory collapse [1, 22, 23]. These features impair concentrating ability and usually give rise to potassium wasting [23]. Distinctions have been made between isolated rise in urea and creatinine and established failure with oliguria, the former usually recovering without filtration and the latter often requiring renal replacement therapy [1, 23].

In this case series, renal involvement was almost universal with 93% of patients having abnormal urinalysis. This is consistent with other studies in which 86% of patients had urine protein excretion of >300 mg/day [24] and proteinuria and pyuria in 67% [20]. Currently, abnormal urinalysis is not part of the diagnostic criteria for leptospirosis, despite being a commonly reported feature in many studies. Given the nonspecific nature of the disease, this represents a useful and inexpensive diagnostic aid.

Biochemically, 63% of cases in this cohort showed a rise in creatinine: 30% had AKI, 30% had reduced urine output, and 33% had hypokalaemia. There was no difference between icteric and anicteric leptospirosis groups regarding renal dysfunction (50% in each group). This was in contrast to previous studies which report jaundice in 80–90% of patients with AKI [25, 26] and renal dysfunction in only 18% of nonjaundiced patients [27, 28]. The combination of jaundice and AKI in severe leptospirosis has historically been referred to as Weil's disease.

Hepatic failure with leptospirosis is rare. Hepatic dysfunction presents with a cholestatic picture and does not usually involve hepatocyte death [9]. This typically manifests with a moderate rise in transaminases and ALP and raised bilirubin. Impaired synthetic function is rare in the absence of multiorgan failure.

The frequency of jaundice in this sample was comparatively low with 40% of patients icteric versus 72–95% in the literature [16, 17]. The presence of jaundice appears to be independent of any of the serious endpoints of AKI, ICU admission, requirement for organ support, and length of hospital stay (Table 4). This is an important finding as previously it has been stated that anicteric leptospirosis has fewer severe consequences than icteric, [1] with icteric leptospirosis cited as having mortality rates of 5–15% [10]. Our data suggest that risk stratification for patients with leptospirosis should not be reliant on the presence of icterus.

### 4.7. Respiratory

Pulmonary involvement in leptospirosis can be severe and is a strong predictor of mortality [1, 10, 14, 21]. It is increasingly recognised as a separate syndrome independent of classically severe leptospirosis, as it is not consistently related to the presence of jaundice [10, 20, 21, 28]. Histologically, there are alveolar infiltrates and haemorrhage suggesting coagulopathy and/or an immunological cause [10]. Some studies have reported pulmonary involvement in nearly 100% of patients while others report it within the range of 20–70% [1, 10]. In this series pulmonary symptoms were uncommon, with 30% reporting cough and 12% shortness of breath. Interestingly, there is often a disassociation between chest X-ray changes and reported symptoms [10]. The small number of cases with pulmonary involvement in this cohort compared to other studies may be in part related to genetically determined host immune responses.

### 4.8. Haematological

Thrombocytopaenia is a common finding in leptospirosis, cited between 50 and 80% [1, 29]. The exact aetiology is not fully understood; possible causes include direct bone marrow toxicity or consumption and immune-mediated response. Derangement of clotting factors has been variably reported. Whether disseminated intravascular coagulation (DIC) is present in leptospirosis is debated; it has been shown inconsistently in animal models but not in humans. There is rarely an effect on fibrinogen, although one study found that nearly 50% of patients had DIC [29]. The rate of haemorrhage in patients with leptospirosis is reported at 23% [29]. The majority of these are mucosal, although there are reports of clinically important gastrointestinal and pulmonary haemorrhage [29].

The presence of thrombocytopaenia is described as an independent predictor of AKI [1] and mortality [10, 29]. In this case series, the presence of thrombocytopaenia was lower than expected at 37%, with no platelet counts less than 50 × 10^9^/L, compared to quoted values of 50–58% [1, 16]. There were only minor derangements in other measures of coagulation in this population. This modest derangement correlates with the lack of pulmonary features and no documented cases of haemorrhage.

### 4.9. Cardiovascular

Hypotension is reported in 60% of patients with leptospirosis [30]. Alongside the classical sepsis picture of inflammatory vasodilation, myocarditis with corresponding reduced cardiac output has been observed in 8% of patients [27, 30]. This may be compounded by failure of renal concentrating ability with polyuria as well as extrarenal losses [24].

In this series, 35% of patients had hypotension (systolic blood pressure <100 mmHg) on admission and 23% required inotropic support during admission. The rates of hypotension and requirement for inotropic support are lower than other studies and are in keeping with the low mortality in New Zealand. Two patients had atrial fibrillation—it is not known if these were pre-existing diagnoses or new and driven by infection.

### 4.10. Outcome

Rates of ICU admission were comparatively low in this cohort, 24% versus 33–64% [31]. Length of hospital stay was short with a mean and median of four days and range 1–9 days, lower than comparable health systems where figures are reported as median 8–10 days and range 1–30 days [31].

The low mortality from leptospirosis in New Zealand is in stark contrast to worldwide trends [3]. Factors influencing this may include that the sv *L. icterohaemorrhagiae,* identified as commonly causing severe leptospirosis, was absent in our series. In our population, features traditionally associated with higher mortality, such as haemorrhage and pulmonary involvement, were uncommon.

The presence of icterus on presentation was not predictive of outcome. Leptospirosis in New Zealand may therefore present as a more homogenous illness and classifying leptospirosis as severe based on the presence of icterus is not justified. The diagnosis of leptospirosis should be considered in the absence of jaundice, and both icteric and anicteric cases were identified and managed promptly in order to prevent deterioration. There may be a role of host immune genetic responses in explaining the different clinical picture in this cohort.

### 4.11. Limitations

Limitations of the study include small sample size and presenting retrospective data up to 2012—a larger review of cases over the past ten years would assist in clarifying the differences observed and allow for statistical analysis. A number of patients had commenced antimicrobial therapy prior to presentation to hospital, which may have impacted reported symptoms. Clinical presentation and radiological and pathological findings vary depending on the length of illness; our cohort was presented on a range of number of days into illness.

## 5. Conclusion

Leptospirosis should be a differential diagnosis in patients presenting with pyrexia, headache, and myalgia. Biochemically, our results suggest that the most useful initial markers to be raised are CRP and proteinuria on urinalysis. These should be contextualised with identified risk factors such as occupation and recreational activities, particularly watersports. Previous literature on cases in New Zealand suggests rash and conjunctival suffusion to be good discriminators of disease, but this study did not find these signs frequently present.

This study has found that previous predictors of severe leptospirosis do not predict any of the outcomes of ICU admission, requirement for organ support, or length of hospital stay. Although this study is not powered to find predictors of outcome, it is clear that anicteric leptospirosis is by no means a benign disease. Further larger studies are required to confirm this difference in predicting the severity of leptospirosis.

## Figures and Tables

**Table 1:** Baseline demographics of patients in study.

Variable	Number (%)
*Age*	
18–30	7 (16.3)
31–50	24 (55.8)
51–70	11 (25.6)
Not given	1 (2.3)

*Gender*	
Male	35 (81.4)
Female	8 (18.6)

*Ethnicity*	
Maori	7 (16.3)
Mixed New Zealand/European	21 (48.8)
Mixed New Zealand/Maori	13 (30.2)
Mixed New Zealand/Maori/European	1 (2.3)
South East Asian	1 (2.3)

*Occupation*	
Meat freezer worker	7 (16.3)
Home kill contractor	1 (2.3)
Meat processor/worker	20 (46.5)
Farmer/cattle worker	12 (27.9)
Horticultural adviser	1 (2.3)
Plasterer	1 (2.3)
Veterinarian	1 (2.3)

**Table 2:** Symptoms and signs reported by patients in comparison with studies in other countries.

Symptom	New Zealand 2012	China 1995 [15] (%)	Hawaii 1998 [16] (%)	Barbados 1990 [17] (%)
	*N* = 43 (%)	*N* = 75	*N* = 353	*N* = 88
Fever	93	—	99	85
Myalgia	86	100	91	49
Headache	86	89	89	76
Nausea	77	56	77	37
Vomiting	60	51	73	50
Anorexia	60	92	82	85
Rigors	53	—	—	—
Arthralgia	40	51	59	21
Conjunctival suffusion	40	97	28	54
Photophobia	30	—	—	5
Diarrhoea	30	30	53	14
Cough	30	55	—	32
Abdominal pain	26	31	51	43
Sore throat	16	—	—	7
Neck stiffness	14	—	27	2
Dyspnoea	12	—	—	—
Rash	12	0	8	2
Jaundice	9	72	—	95

*Sign*				
Abdominal tenderness	42	—	—	25
Oliguria	30	—	—	13
Pulmonary crepitations	21	—	17	—
Hepatomegaly	12	—	16	27
Meningism	7	—	27	—
Lymphadenopathy	2	—	—	25
Splenomegaly	2	—	4	—

**Table 3:** Laboratory abnormalities in comparison with Hawaii data [17].

Sample measured	Range	New Zealand percentage abnormal (%)	Hawaii percentage abnormal (%)
WCC	—	25	39
Platelet count	77–144	37	58
CRP	80–306	100	—
Creatinine	117–912	63	54
Urea	8–33	56	49
Bilirubin	23–76	40	70
ALT	42–505	67	73
APTT	24–40	33	—
pH	7.23–7.33	37	—
CK	291–430	36	—
Haematuria	—	74	72
Proteinuria	—	88	54

WCC = white cell count, CRP = C-reactive protein, ALT = alanine aminotransferase, APTT = activated partial thromboplastin time, CK = creatinine kinase.

**Table 4:** Length of stay and features of severity in icteric and anicteric patients.

	Length of hospital admission (days)		ICU admission [%]	AKI [%]	Required haemofiltration [%]	Required vasopressor support [%]
	Mean	Range				
Icteric *n* = 17	4	1–7	4 [23]	5 [29]	0 [0]	3 [18]
Anicteric *n* = 26	4	1–9	8 [30]	8 [31]	1 [4]	7 [27]
Total *n* = 43	4	1–9	12 [28]	13 [30]	1 [2]	10 [23]

## Data Availability

Anonymised data are available on request from the corresponding author.
